# Field Balancing and Harmonic Vibration Suppression in Rigid AMB-Rotor Systems with Rotor Imbalances and Sensor Runout

**DOI:** 10.3390/s150921876

**Published:** 2015-08-31

**Authors:** Xiangbo Xu, Shao Chen

**Affiliations:** School of Technology, Beijing Forestry University, No.35 Tsinghua East Road, Haidian District, Beijing 100083, China; E-Mail: chenshao1019@163.com

**Keywords:** Active magnetic bearing, field balancing, vibration suppression, rotor imbalances, sensor runout

## Abstract

Harmonic vibrations of high-speed rotors in momentum exchange devices are primary disturbances for attitude control of spacecraft. Active magnetic bearings (AMBs), offering the ability to control the AMB-rotor dynamic behaviors, are preferred in high-precision and micro-vibration applications, such as high-solution Earth observation satellites. However, undesirable harmonic displacements, currents, and vibrations also occur in the AMB-rotor system owing to the mixed rotor imbalances and sensor runout. To compensate the rotor imbalances and to suppress the harmonic vibrations, two control methods are presented. Firstly, a four degrees-of-freedom AMB-rotor model with the static imbalance, dynamic imbalance, and the sensor runout are described. Next, a synchronous current reduction approach with a variable-phase notch feedback is proposed, so that the rotor imbalances can be identified on-line through the analysis of the synchronous displacement relationships of the geometric, inertial, and rotational axes of the rotor. Then, the identified rotor imbalances, which can be represented at two prescribed balancing planes of the rotor, are compensated by discrete add-on weights whose masses are calculated in the vector form. Finally, a repetitive control algorithm is utilized to suppress the residual harmonic vibrations. The proposed field balancing and harmonic vibration suppression strategies are verified by simulations and experiments performed on a control moment gyro test rig with a rigid AMB-rotor system. Compared with existing methods, the proposed strategies do not require trial weights or an accurate model of the AMB-rotor system. Moreover, the harmonic displacements, currents, and vibrations can be well-attenuated simultaneously.

## 1. Introduction

Extremely high pointing stability is required in high-resolution Earth observation satellites, which are equipped with many sensitive payloads [1]. However, the pointing stability is severely affected by undesirable harmonic vibrations of rapidly spinning rigid rotors in momentum exchange devices, such as flywheels and control moment gyros [2,3]. These harmonic vibrations are mainly caused by the rotor mass imbalance and imperfect bearing properties [4]. If conventional ball bearings are adopted to support the rotor, the bearing stiffness varies periodically, and then multiple vibrations will be induced and be transferred to the spacecraft directly [5]. To suppress these undesirable harmonic vibrations, additional special isolation devices are usually designed [6]. Active magnetic bearings (AMBs) offer many advantages of contact-free levitation, small noise emission and, especially, active controlability [7,8]. In contrast with the conventional ball bearings, AMBs can employ field balancing and vibration suppression methods without adding any hardware. Hence, the AMBs are preferred in micro-vibration applications [9]. 

The rotor imbalances, which result from lack of alignment between the inertial axis of the rotor and its geometric axis, are the dominant causes of synchronous vibrations. Balancing is a process of improving the mass distribution of the rotor, so that the inertial axis coincides with the geometric axis. The main balancing methods can be divided into three categories: balancing with a special machine, automatic balancing with a self-compensating device, and field balancing. The first method is usually employed by manufacturers with a balancing machine before installation [10]. Only a mediocre level of balancing accuracy can be achieved due to measurement precision, limited rotational speed, structural resonance, and various disturbances. The widely-used self-compensation balancing device is a ball balancer [11,12]. The main advantage of this method is its ability of compensating variable imbalances during operation. However, it is acceptable to add a balancing device for control moment gyros with an inner rotor structure and limited space. Field balancing is a method of balancing a rotor in its own supporting structure rather than in a balancing machine. The imbalances can be reduced to a relatively low level because the balancing process can be completed in the actual working condition of the rotor. The influence coefficient method is effective [13], yet too many trials will prolong the balancing process. The number of the trials can be reduced if the unknown imbalances are identified through vibration responses and precise modeling of the rotor-bearing system [14]. However, if a direct identification is performed without effective control of the AMB-rotor system, the identification precision is highly dependent on the accuracy of the system model. As one of the most important features, AMBs can modulate the dynamic behaviors of the rotor by supplying active real-time control. A more accurate identification can be realized if the rotation axis of the rotor can be properly controlled in a certain position [15]. The field balancing can be achieved by adding discrete weights, once the rotor imbalance identification is completed. It is suitable for rigid rotors because the rotor imbalances change little over the full range of the rotor speed.

To control the rotation axis of the rotor, traditional methods fall into two categories. In the former one, synchronous control force and torque are provided for forcing the rotor to rotate around its geometric axis. The imbalances can be identified through on-line estimations or well-established dynamic knowledge from the control force and torque to the rotor imbalances [16]. However, the control effect is limited by the bandwidth of the AMB-rotor system and the synchronous control voltage that can be supplied, so it is not suitable for a high-speed rotor driven by a low-voltage power source [17]. An approach for solving this problem is the latter one, which controls the rotor to spin around its inertial axis [18]. The imbalance compensator method in the AMB system is firstly proposed by Habermann, a so-called automatic balancing system is proposed to eliminate the synchronous current and to reduce the housing vibration in [19]. In fact, precise control current should be generated to compensate the vibration caused by the displacement stiffness [20]. However, the precision of this approach mainly depends on the parameter accuracy of the controlled object [21], especially the voltage-source power amplifiers, whose performances vary greatly with many parameters, such as temperature and the inductance of the AMB coil [22]. The precision of rotation around the inertial axis will inevitably decrease due to the inaccurate control current caused by the errors and variations of the power amplifiers [23]. Instead, a synchronous current reduction approach is practically preferred because of low copper loss and, especially, structural stability [21]. To reduce the synchronous current, a notch filter is widely used and studied owing to its advantages of low computation effort and easy analysis of closed-loop stability [24,25]. However, residual synchronous currents, which are generated by the motion induced voltage, will still remain if the synchronous control voltage is merely cleaned in the AMB-rotor system with voltage-source power amplifiers [26]. 

Position of the rotation axis is dependent on the rotor speed when the synchronous current reduction is achieved and then further identification of the imbalances, according to the measured synchronous displacements, can be performed. However, the sensor runout, which originates from nonuniform electrical and magnetic properties around the sensing surface of the rotor, brings noise at first and multiple harmonics of the rotor speed to the measured displacements [27]. Furthermore, the first harmonic of the sensor runout is mixed with the rotor imbalances. Hence, errors will occur if the synchronous measured displacements are directly employed to estimate the rotor imbalances [28]. For the control moment gyro in high-resolution Earth observation satellites, the undesirable harmonic vibrations induced by the rotor imbalances and the sensor runout have to be effectively suppressed. Setiawan *et al.* did comprehensive research on an adaptive compensation approach whose stability is guaranteed by a Lyapunov function [29,30]. However, a higher robustness of the controller needs complex design and extensive computational effort. To solve this problem, Xu proposed a harmonic disturbance rejection method with a repetitive controller [31]. However, the repetitive control method did not separate the rotor imbalance from the sensor runout, but just reduced all the harmonic currents. Unlike sensor runout, which is essentially sensor noise and only generates vibration through the current stiffness of the AMB, the rotor imbalances are due to uneven mass distribution and can cause vibrations through both the current stiffness and the displacement stiffness [31]. Hence, the synchronous vibrations caused by the rotor imbalance and the displacement stiffness still remain, if only the repetitive control method is employed without balancing the rotor.

In this study, the rotor imbalances are identified through a synchronous current reduction method with a variable-phase notch feedback. Next, the static and dynamic imbalances are presented as two imbalanced masses located in two prescribed balancing planes of the rotor, and add-on discrete weights with the masses’ values calculated in the vector form are used to compensate the rotor imbalances. Then, the residual harmonic vibrations induced by the sensor runout and the current stiffness are suppressed by only reducing the harmonic currents with a repetitive control algorithm. Finally, the effectiveness of the field balancing and harmonic vibration suppression strategies are demonstrated by simulations and experiments performed on a control moment gyro test rig with a high-speed rigid AMB-rotor system. 

## 2. Modeling of the AMB-Rotor System

**Figure 1:**
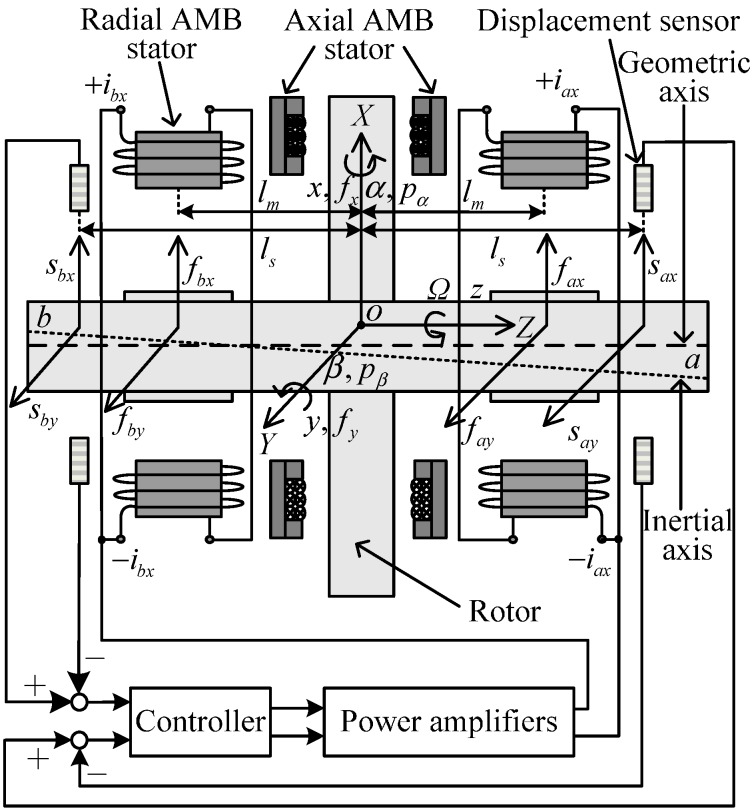
Diagram of the AMB-rotor system in the *x*-*z* plane.

The AMB-rotor system, whose block diagram in the *x*-*z* plane is shown in Figure 1, consists of an imbalanced rotor, radial AMB stators, axial AMB stators, displacement sensors, controller, and power amplifiers. *o* is the center of the AMB stators and the origin of the coordinate *XYZ*. *l_m_* and *l_s_* are the distances from *o* to the centers of a radial AMB stator and a displacement sensor, respectively. The rotor has six degrees-of-freedom (DOF): three translational motions with displacements of *x*, *y*, *z*, and three rotational motions with two radial displacements of *α*, *β*, and one axial rotor speed of *Ω*. *Ω* is driven by a rotor motor and is independent of the AMB-rotor system. Moreover, *z* is controlled by the axial AMBs, which have little coupling with the radial AMBs and the imbalances.

Consequently, we only focus on the radial 4-DOF directions (*x*, *y*, *α*, *β*) controlled by the radial AMBs. Only two pairs of radial AMBs are shown in Figure 1, as the other two pairs are oriented orthogonal to the paper plane. *a* and *b* denote the two terminals of the rotor. Signals of the AMB-rotor system are defined in three coordinates: the sensor coordinate, the stator coordinate, and the generalized coordinate. According to the measured displacements (*s_ax_*, *s_bx_*, *s_ay_*, and *s_by_* in the four decentralized sensor directions of *ax*, *bx*, *ay*, and *by*, respectively) of the geometric axis by the displacement sensors, the controller drives the power amplifiers to generate four coil currents *i_ax_*, *i_bx_*, *i_ay_*, and *i_by_* (only *i_ax_* and *i_bx_* of the shown radial AMB stators are visible in Figure 1). Finally, control forces of *f_ax_*, *f_bx_*, *f_ay_*, and *f_by_* in the four decentralized stator directions are produced to levitate the rotor in the radial 4-DOF directions. The generalized displacement and force vectors are defined as [*x β y −α*]^T^ and [*f_x_ p_β_ f_y_ −p_α_*]^T^ in the generalized coordinate. Then, the displacements of the geometric and inertial axes can be expressed as ***q****_G_* = [ *x_G_ β_G_ y_G_ −α_G_*]^T^ and ***q**_I_* = [ *x_I_ β_I_ y_I_ −α_I_*]^T^, respectively. Since the static and dynamic imbalances are the eccentricity and inclination angle of ***q****_G_* and ***q****_I_*, the imbalance vector Δ***q*** is defined in the generalized coordinate:

(1) $$\Delta q=\left[\begin{array}{c} \Delta x\\ \Delta \beta \\ \Delta y\\ -\Delta \alpha \end{array}\right]=q_{I}-q_{G}=\left[\begin{array}{c} \varepsilon \cos\left(\Omega t+\chi \right)\\ \sigma \sin\left(\Omega t+\delta \right)\\ \varepsilon \sin\left(\Omega t+\chi \right)\\ -\sigma \cos\left(\Omega t+\delta \right) \end{array}\right]$$

where *ε* and *χ* are the amplitude and the initial phase of the static imbalance, respectively; *σ* and *δ* are the amplitude and the initial phases of the dynamic imbalance, respectively. 

The sensor runout is the noise of the displacement sensor, and its vector ***q****_sr_* is defined in the sensor coordinate:

(2) $$q_{sr}=\left[\begin{array}{c} s_{rax}\\ s_{rbx}\\ s_{ray}\\ s_{rby} \end{array}\right]=\left[\begin{array}{c} \sum_{i=1}^{n}s_{asi}\sin(i\Omega t+\alpha_{si})\\ \sum_{i=1}^{n}s_{bsi}\sin(i\Omega t+\beta_{si})\\ \sum_{i=1}^{n}s_{asi}\sin(i\Omega t+\alpha_{si}-\frac{i\pi }{2})\\ \sum_{i=1}^{n}s_{bsi}\sin(i\Omega t+\beta_{si}-\frac{i\pi }{2}) \end{array}\right]$$

where *s_rax_*, *s_rbx_*, *s_ray_*, and *s_rby_* are the sensor runout in the *ax*, *bx*, *ay*, and *by* channels of the sensor coordinate, *n* is the harmonic number, *s_asi_* and *s_bsi_* are the harmonic Fourier coefficients, and *α_si_* and *β_si_* are the harmonic initial phases.

Assuming that the parameters among the four decentralized directions have identical values and the gravity is acting in the *Z* direction, the dynamics of the rotor in the radial 4-DOF directions can be given based on earlier work [30]:

(3) $$\begin{array}{c} \left(Ms^{2}+Gs\right)\left[q_{G}\left(s\right)+\Delta q\left(s\right)\right]=k_{x}T_{ft}q_{G}\left(s\right)\\ -k_{i}\left\{G_{w}(s)T_{fs}G_{s}(s)\left[q_{G}\left(s\right)+{T_{s}}^{-1}q_{sr}\left(s\right)\right]+k_{v}G_{w}(s)T_{ft}q_{G}\left(s\right)\right\} \end{array}$$

where ***M*** is the mass matrix, ***G*** is the gyroscopic matrix, *k_i_* is the current stiffness, *G_w_*(*s*) is the transfer function of the power amplifier, *k_v_* is a coefficient related to the motion induced voltage, *T_fs_*, *T_s_*, and *T_ft_* are coordinate transfer matrices, *G_s_*(*s*) is the displacement controller matrix designed in the generalized coordinate, and *k_x_* is the displacement stiffness:

$$M=\left[\begin{array}{cccc} m & 0 & 0 & 0\\ 0 & J_{r} & 0 & 0\\ 0 & 0 & m & 0\\ 0 & 0 & 0 & J_{r} \end{array}\right]\;G=\left[\begin{array}{cccc} 0 & 0 & 0 & 0\\ 0 & 0 & 0 & J_{z}\Omega \\ 0 & 0 & 0 & 0\\ 0 & -J_{z}\Omega & 0 & 0 \end{array}\right]\;T_{s}=k_{s}\left[\begin{array}{cccc} 1 & l_{s} & 0 & 0\\ 1 & -l_{s} & 0 & 0\\ 0 & 0 & 1 & l_{s}\\ 0 & 0 & 1 & -l_{s} \end{array}\right]$$

$$G_{w}(s)=k_{w}\frac{\omega_{w}}{s+\omega_{w}}\;T_{fs}=\text{diag}\left[2k_{s}\;2k_{s}l_{m}l_{s}\;2k_{s}\;2k_{s}l_{m}l_{s}\right]\;T_{ft}=\text{diag}\left[2\;2{l_{m}}^{2}\;2\;2{l_{m}}^{2}\right]$$

$$G_{s}\left(s\right)=\left(k_{P}+\frac{k_{I}}{s}+k_{D}s\right)I_{4}+\left[\begin{array}{cccc} 0 & 0 & 0 & 0\\ 0 & \frac{k_{rh}\Omega s}{s+\omega_{rh}}\cos\phi -\frac{k_{rl}\Omega \omega_{rl}}{s+\omega_{rl}}\cos\varphi & 0 & -\frac{k_{rh}\Omega s}{s+\omega_{rh}}\sin\phi +\frac{k_{rl}\Omega \omega_{rl}}{s+\omega_{rl}}\sin\varphi \\ 0 & 0 & 0 & 0\\ 0 & \frac{k_{rh}\Omega s}{s+\omega_{rh}}\sin\phi -\frac{k_{rl}\Omega \omega_{rl}}{s+\omega_{rl}}\sin\varphi & 0 & \frac{k_{rh}\Omega s}{s+\omega_{rh}}\cos\phi -\frac{k_{rl}\Omega \omega_{rl}}{s+\omega_{rl}}\cos\varphi \end{array}\right]$$

where *m* is the rotor mass, *J_r_* and *J_z_* are the transverse and polar moments of inertia of the rotor, respectively, *k_s_* is the coefficient of the displacement sensor, *k_P_* and *k_D_* are the coefficients of the typical proportional-derivative controller, a pseudo integrator is used to avoid over-restriction, *k_I_* and *k_IM_* are its parameters, ***I****_4_* is a 4 × 4 unit matrix, *k_rh_* and *k_rl_* are gains of the cross feedback control, *ω_rh_* and *ω_rl_* are the cutoff angular frequencies of the high-pass and low-pass filters, respectively, *ϕ* and *φ* are the cross phases, *k_w_* and *ω_w_* are the gain and the cutoff angular frequency of the simplified low-pass power amplifier model. The PID controller is employed to stabilize the AMB system with a negative stiffness, while the cross feedback controller is designed to suppress the gyroscopic effect [26].

The AMB-rotor model can be divided into two uncoupled subsystems: one is related to the translational motions which are also uncoupled in the *X* and *Y* directions, and the other is related to the two coupled rotational motions:

(4) $$\left\{ \begin{array}{l} ms^{2}\left[x_{G}\left(s\right)+\Delta x\left(s\right)\right]=\left\{2k_{x}-2k_{i}k_{s}G_{w}\left(s\right)\left[C_{t}\left(s\right)+k_{Dv}s\right]\right\}x_{G}\left(s\right)-2k_{i}G_{w}\left(s\right)C_{t}\left(s\right)x_{sr}\left(s\right)\\ ms^{2}\left[y_{G}\left(s\right)+\Delta y\left(s\right)\right]=\left\{2k_{x}-2k_{i}k_{s}G_{w}\left(s\right)\left[C_{t}\left(s\right)+k_{Dv}s\right]\right\}y_{G}\left(s\right)-2k_{i}G_{w}\left(s\right)C_{t}\left(s\right)y_{sr}\left(s\right) \end{array}\right.$$

(5) $$\left\{ \begin{array}{l} J_{r}s^{2}\left[\alpha_{G}\left(s\right)+\Delta \alpha \left(s\right)\right]+J_{z}\Omega s\left[\beta_{G}\left(s\right)+\Delta \beta \left(s\right)\right]=C_{rc}\left(s\right)\beta_{G}\left(s\right)-2k_{i}l_{m}G_{w}\left(s\right)C_{rs}\left(s\right)\alpha_{sr}\\ +2k_{i}k_{s}l_{m}l_{s}G_{w}\left(s\right)+\left\{2k_{x}{l_{m}}^{2}-2k_{i}k_{s}l_{m}l_{s}G_{w}\left(s\right)\left[C_{rs}\left(s\right)+\frac{l_{m}}{l_{s}}k_{Dv}s\right]\right\}\alpha_{G}\left(s\right)\\ +2k_{i}l_{m}G_{w}\left(s\right)C_{rc}\left(s\right)\beta_{sr}\\ J_{r}s^{2}\left[\beta_{G}\left(s\right)+\Delta \beta \left(s\right)\right]-J_{z}\Omega s\left[\alpha_{G}\left(s\right)+\Delta \alpha \left(s\right)\right]=-2k_{i}k_{s}l_{m}l_{s}G_{w}\left(s\right)C_{rc}\left(s\right)\alpha_{G}\left(s\right)\\ +\left\{2k_{x}{l_{m}}^{2}-2k_{i}k_{s}l_{m}l_{s}G_{w}\left(s\right)\left[C_{rs}\left(s\right)+\frac{l_{m}}{l_{s}}k_{Dv}s\right]\right\}\beta_{G}\left(s\right)-2k_{i}l_{m}G_{w}\left(s\right)C_{rs}\left(s\right)\beta_{sr}\\ -2k_{i}l_{m}G_{w}\left(s\right)C_{rc}\left(s\right)\alpha_{sr} \end{array}\right.$$

where:

$$\left\{ \begin{array}{l} \Delta x\left(s\right)=\varepsilon \frac{\cos\chi s-\Omega \sin\chi }{s^{2}+\Omega^{2}}\\ \Delta y\left(s\right)=\varepsilon \frac{\sin\chi s+\Omega \cos\chi }{s^{2}+\Omega^{2}}\\ \Delta \alpha \left(s\right)=\sigma \frac{\cos\delta s-\Omega \sin\delta }{s^{2}+\Omega^{2}}\\ \Delta \beta \left(s\right)=\sigma \frac{\sin\delta s+\Omega \cos\delta }{s^{2}+\Omega^{2}} \end{array}\right.\;\{\begin{array}{l} k_{Dv}=k_{v}/k_{s}\\ C_{t}\left(s\right)=k_{P}+\frac{k_{I}}{s+k_{IM}}+k_{D}s\\ C_{rs}\left(s\right)=k_{P}+\frac{k_{I}}{s+k_{IM}}+k_{D}s+\frac{k_{rh}\Omega s\cos\phi }{s+\omega_{rh}}-\frac{k_{rl}\Omega \omega_{rl}\cos\varphi }{s+\omega_{rl}}\\ C_{rc}\left(s\right)=\frac{k_{rh}\Omega s}{s+\omega_{rh}}\sin\phi -\frac{k_{rl}\Omega \omega_{rl}}{s+\omega_{rl}}\sin\varphi \end{array}\;\{\begin{array}{l} x_{sr}=\frac{s_{rax}+s_{rbx}}{2}\\ \beta_{sr}=\frac{s_{rax}-s_{rbx}}{2}\\ y_{sr}=\frac{s_{ray}+s_{rby}}{2}\\ \alpha_{sr}=\frac{s_{rby}-s_{ray}}{2} \end{array}$$

As shown in Equations (3)–(5), the measured displacements of the geometric axis expressed in the generalized coordinate is $q_{G}+{T_{s}}^{-1}q_{sr}$ (defined as $q_{GM}={\left[x_{GM}\;\beta_{GM}\;y_{GM}\;-\alpha_{GM}\right]}^{T}$), according to which *G_s_*(*s*) controls the rotor. The static and dynamic imbalances induce synchronous vibration force and torque through *C_t_*(*s*), *k_v_*, and *k_x_* in the translational and rotational motions, respectively, whereas the sensor runout causes first and multiple harmonic vibration force and torque only through *C_t_*(*s*) due to its nature of the displacement sensor noises. To achieve field balancing and harmonic vibration suppression, identification of Δ***q*** from ***q****_GM_* is primary.

The output force and torque from AMB are functions of the coil current and the geometric displacement respectively related to *k_i_* and *k_x_* [30]. If a reduction of the synchronous currents can be achieved, only the force and torque related to *k_x_* remain, and Equation (3) will be given by:

(6) $$\left(Ms^{2}+Gs\right)\left[q_{G}\left(s\right)+\Delta q\left(s\right)\right]=k_{x}T_{ft}q_{G}\left(s\right)$$

The synchronous displacement relationship of Δ***q*** and ***q****_G_* can be easily determined. Then, a method of identifying Δ***q*** from the noisy ***q****_GM_* is designed.

## 3. Design of the Synchronous Current Reduction Controller

**Figure 2:**
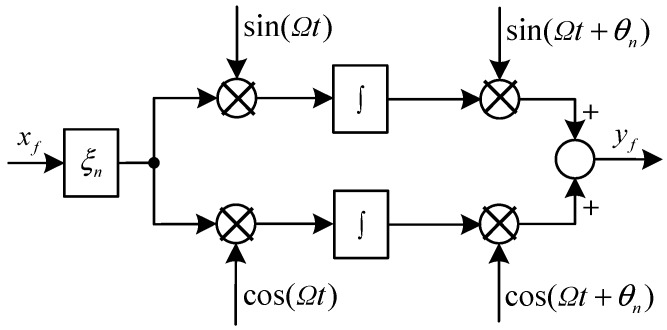
Diagram of the improved notch feedback block.

A notch feedback block [32], which has an infinite gain at the synchronous frequency, is designed to reduce the synchronous current. The diagram of the notch feedback block is shown in Figure 2, where *x_f_* is the input signal with a component at the frequency of *Ω* to be separated, *ξ_n_* is the damping coefficient, *θ_n_* is the phase shift, *y_f_* is the output signal. The dynamic equation of the internal feedback block can be expressed as:

(7) $$y_{f}=\xi_{n}\left[\begin{array}{cc} \sin\left(\Omega t+\theta_{n}\right) & \cos\left(\Omega t+\theta_{n}\right) \end{array}\right]\int \left[\begin{array}{c} x_{f}\sin\left(\Omega t\right)\\ x_{f}\cos\left(\Omega t\right) \end{array}\right]dt$$

We can easily verify the following differential equation:

(8) $${\ddot{y}}_{f}+\Omega^{2}y_{f}=\xi_{n}\left({\dot{x}}_{f}\cos\theta_{n}-x_{f}\Omega \sin\theta_{n}\right)$$

Consequently, the transfer function of the developed notch feedback block can be obtained as:

(9) $$C_{nf}\left(s\right)=\frac{y_{f}\left(s\right)}{x_{f}\left(s\right)}=\xi_{n}\frac{s\cos\theta_{n}-\Omega \sin\theta_{n}}{s^{2}+\Omega^{2}}$$

**Figure 3:**
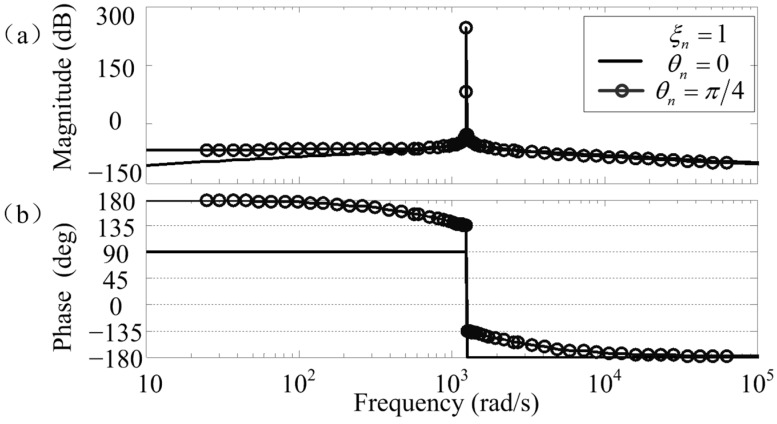
Bode diagrams of the improved notch feedback block: (**a**) magnitude; (**b**) phase.

Bode diagrams of *C_nf_*(*s*) are shown in Figure 3, where *Ω* = 400*π* rad/s, *ξ_n_* = 1, and values of *θ_n_* are chosen as 0 and π/4 to make comparisons. As can be seen, the magnitude at the notch frequency of *Ω* is infinite, and this can also be verified by using Equation (9). Therefore, a notch filter with a notch frequency of *Ω* can be formed if *C_nf_*(*s*) is used as a feedback element [23]. Furthermore, the phase at the notch frequency is *θ_n_*. Hence, design and stability analysis of *C_nf_*(*s*) can be simplified, because the synchronous phase shift can be easily set.

**Figure 4:**
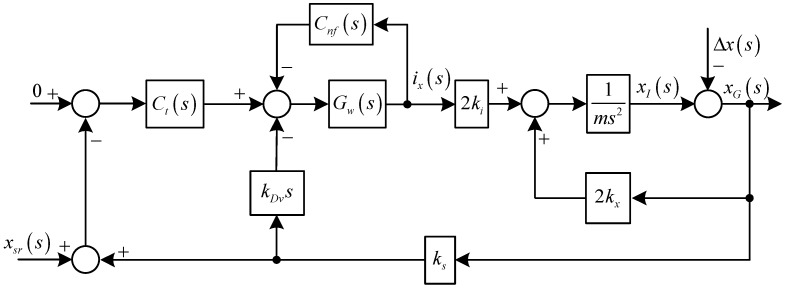
Diagram of the translational subsystem in the *X* direction with the notch feedback.

A generalized current vector ***i*** is defined as [30]:

$$i={\left[i_{x}\;i_{\beta }\;i_{y}\;-i_{\alpha }\right]}^{T}\text{=}\frac{1}{2}{\left[i_{ax}\text{+}i_{bx}\;i_{ax}-i_{bx}\;i_{ay}\text{+}i_{by}\;i_{ay}-i_{by}\right]}^{T}$$

The variable-phase notch feedback block is incorporated into the AMB-rotor control system to achieve a synchronous infinite-gain feedback of ***i***. Since the translational motions in the *X* and *Y* directions are symmetrical, only the controller design in the *X* direction is introduced, and the diagram is shown in Figure 4. 

From Figure 4, we have the expression of *i*_x_(*s*) as:

(10) $$i_{x}\left(s\right)=C_{i1}\left(s\right)\Delta x\left(s\right)+C_{i2}\left(s\right)x_{sr}\left(s\right)$$

where:

$$C_{i1}\left(s\right)=\frac{ms^{2}k_{s}\left[k_{Dv}s+C_{t}\left(s\right)\right]G_{w}\left(s\right)}{\left(ms^{2}-2k_{x}\right)\left[1+G_{w}\left(s\right)C_{nf}\left(s\right)\right]\text{+}2k_{i}k_{s}\left[k_{Dv}s+C_{t}\left(s\right)\right]G_{w}\left(s\right)}$$

$$C_{i2}\left(s\right)=-\frac{C_{t}\left(s\right)G_{w}\left(s\right)\left(ms^{2}-2k_{x}\right)}{\left(ms^{2}-2k_{x}\right)\left[1+G_{w}\left(s\right)C_{nf}\left(s\right)\right]\text{+}2k_{i}k_{s}\left[k_{Dv}s+C_{t}\left(s\right)\right]G_{w}\left(s\right)}$$

It is easy to verify the following equation:

(11) $${C_{i1}\left(s\right)|}_{s=j\Omega }={C_{i2}\left(s\right)|}_{s=j\Omega }=0$$

Hence, the synchronous *i*_x_(*s*) will be cleaned, only if the stability can be guaranteed. The poles of the closed-loop system in Figure 4 are the roots of the following equation:

(12) $$\left(ms^{2}-2k_{x}\right)\left[1+G_{w}\left(s\right)C_{nf}\left(s\right)\right]\text{+}2k_{i}k_{s}\left[k_{Dv}s+C_{t}\left(s\right)\right]G_{w}\left(s\right)=0$$

**Figure 5:**
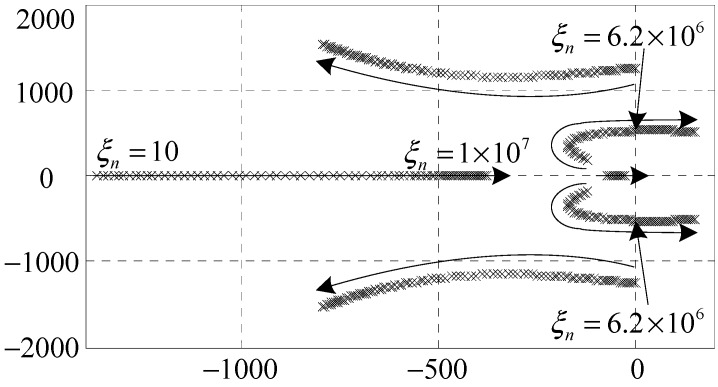
Root locus of the clean synchronous *i*_x_ control subsystem when *θ_n_* = 0.3*π*, *ξ_n_* ∈ [10, 1 × 10^7^].

**Figure 6:**
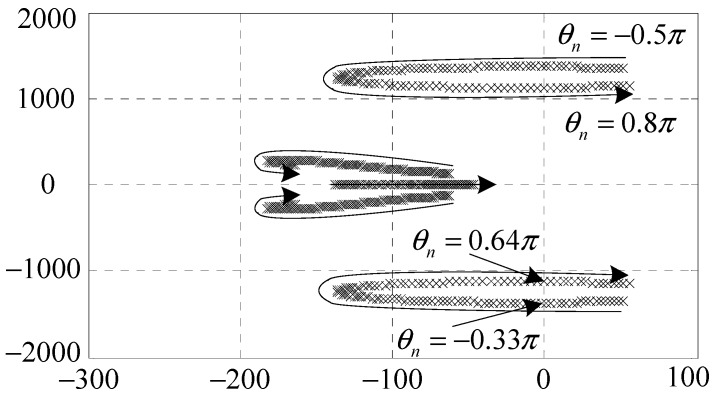
Root locus of the clean synchronous *i*_x_ control subsystem when *ξ_n_* = 2 × 10^6^, *θ_n_* ∈ [−0.5*π*, 0.8*π*].

*C_nf_*(*s*) may endanger the stability of the AMB system. A root locus method is employed to analyze the stability and determine the values of the parameters in *C_nf_*(*s*). As shown in Figure 5 and Figure 6, the stable value ranges of *ξ_n_* and *θ_n_* are [0, 6.2 × 10^6^] and [−0.33*π*, 0.64*π*], respectively.

**Figure 7:**
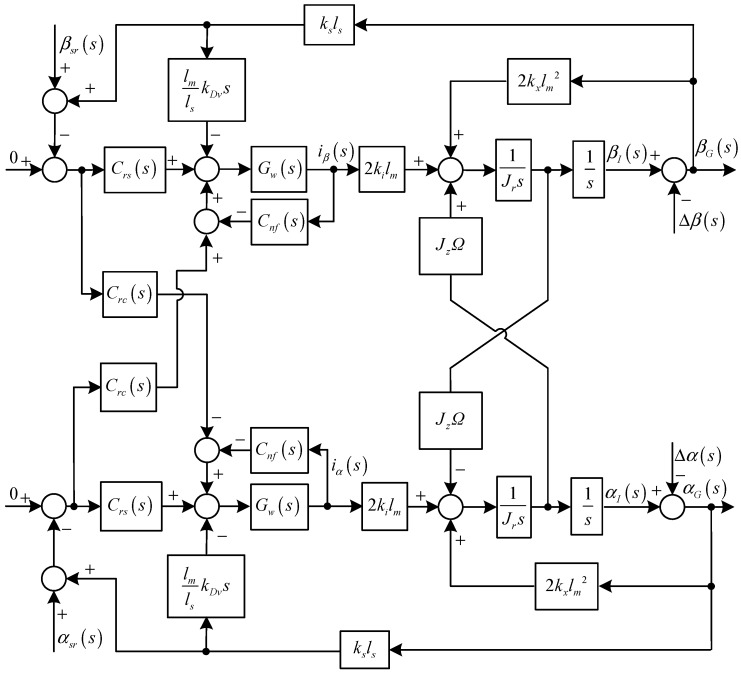
Diagram of the rotational subsystem with two variable-phase notch feedback blocks.

A similar control and analysis method can be utilized to achieve the synchronous current reduction in the rotational subsystem. However, the rotational motions in the *α* and *β* directions are coupled due to the gyroscopic effect, so two notch feedback blocks are designed in the rotational subsystem, whose diagram is shown in Figure 7. 

## 4. Identification of the Rotor Imbalances and Field Balancing

To analyze the dynamics of the AMB-rotor system upon reduction of the synchronous currents, let:

(13) $$\{\begin{array}{l} \begin{array}{l} r_{G}=x_{G}+jy_{G}\\ \Delta r=\Delta x+j\Delta y\\ r_{GM}=x_{GM}+jy_{GM} \end{array}\\ \begin{array}{l} {ο}_{G}=\alpha_{G}+j\beta_{G}\\ \Delta ο=\Delta \alpha +j\Delta \beta \\ {ο}_{GM}=\alpha_{GM}+j\beta_{GM} \end{array} \end{array}$$

where *j* is the complex unit. Then Equation (6) can be simplified as follows:

(14) $$\left\{ \begin{array}{l} ms^{2}\left[r_{G}\left(s\right)+\Delta r\left(s\right)\right]=2k_{x}r_{G}\left(s\right)\\ \left(J_{r}s^{2}-jJ_{z}\Omega s\right)\left[o_{G}\left(s\right)+\Delta ο\left(s\right)\right]=2k_{x}{l_{m}}^{2}o_{G}\left(s\right) \end{array}\right.$$

where:

$$\{\begin{array}{l} \Delta r\left(s\right)=\frac{\varepsilon e^{j\chi }}{s-j\Omega }\\ \Delta ο\left(s\right)=\frac{\sigma e^{j\delta }}{s-j\Omega } \end{array}$$

From Equation (14), the synchronous displacements of the geometric axis can be formulated as:

(15) $$\left\{ \begin{array}{l} {r_{G}\left(s\right)|}_{s=j\Omega }\text{=}-\frac{m\Omega^{2}}{m\Omega^{2}+2k_{x}}\varepsilon e^{j\chi }\\ {o_{G}\left(s\right)|}_{s=j\Omega }=-\frac{\left(J_{r}-J_{z}\right)\Omega^{2}}{\left(J_{r}-J_{z}\right)\Omega^{2}+2k_{x}{l_{m}}^{2}}\sigma e^{j\delta } \end{array}\right.$$

**Figure 8:**
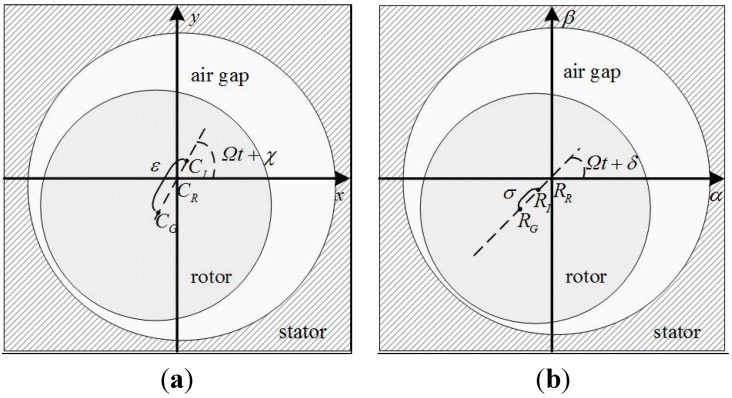
The synchronous displacement relationships of the geometric, inertial, and rotational axes: (**a**) translation; (**b**) rotation.

The synchronous displacement relationships of the geometric, inertial, and rotational axes of the rotor are shown in Figure 8, where *C_G_*, *C_I_*, and *C_R_* are the centers of the geometric, inertial, and rotational axes, *R_G_*, *R_I_*, and *R_R_* are the angles of the geometric, inertial, and rotational axes, respectively. Then we have:

(16) $$\left\{ \begin{array}{l} \left|C_{G}{\text{C}}_{R}\right|\text{=}\frac{m\Omega^{2}}{m\Omega^{2}+2k_{x}}\varepsilon \\ \left|R_{G}{\text{R}}_{R}\right|=\left|\frac{\left(J_{r}-J_{z}\right)\Omega^{2}}{\left(J_{r}-J_{z}\right)\Omega^{2}+2k_{x}{l_{m}}^{2}}\right|\sigma \end{array}\right.$$

It is noted that the rotor of the control moment gyro is designed to be flat ($J_{z}\approx 1.32\;J_{r}$) for a large moment of inertia. It can be regarded as peer rigid because the first bending-critical speed (887 Hz) is far beyond the nominal speed (200 Hz). 

**Figure 9:**
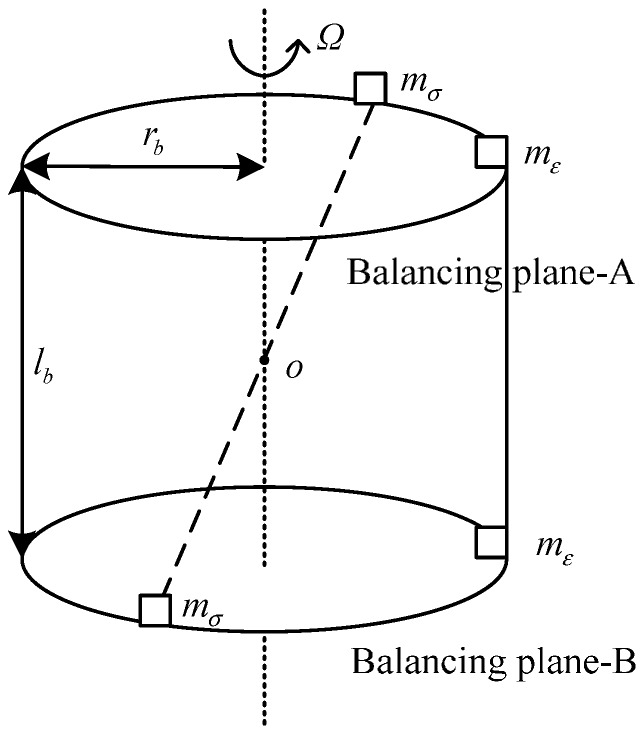
The schematic diagram of the double-plane balancing method.

The synchronous ***q****_GM_* consists of the synchronous ***q****_G_* and the first harmonic of ***q****_sr_*. From Equations (2) and (16), the amplitude of the synchronous ***q****_G_* is related to *Ω*, whereas the amplitude and the initial phase is not related to *Ω*. Hence, a two-speed separation method can be used to identify Δ***q***. The displacement sensors and the angular-position sensor are used to measure a rotor revolution of ***q****_GM_* and its relative phase to the reference point. Then ***q****_sr_* can be cancelled with the difference of ***q****_GM_* at two varied speeds of *Ω_1_* and *Ω_2_*, which leads to:

(17) $$\left\{ \begin{array}{l} \Delta \left|r_{GM}\right|=\Delta \left|C_{G}{\text{C}}_{R}\right|=\left(\frac{m{\Omega_{2}}^{2}}{m{\Omega_{2}}^{2}+2k_{x}}-\frac{m{\Omega_{1}}^{2}}{m{\Omega_{1}}^{2}+2k_{x}}\right)\varepsilon \\ \Delta \left|o_{GM}\right|=\Delta \left|R_{G}{\text{R}}_{R}\right|=\left(\left|\frac{\left(J_{r}-J_{z}\right){\Omega_{2}}^{2}}{\left(J_{r}-J_{z}\right){\Omega_{2}}^{2}+2k_{x}{l_{m}}^{2}}\right|-\left|\frac{\left(J_{r}-J_{z}\right){\Omega_{1}}^{2}}{\left(J_{r}-J_{z}\right){\Omega_{1}}^{2}+2k_{x}{l_{m}}^{2}}\right|\right)\sigma \end{array}\right.$$

*ε* and *σ* can be calculated from Equation (17) by using the displacement sensors to measure $\left|C_{G}C_{R}\right|$, $\left|R_{G}R_{R}\right|$ and the angular-position sensor to measure *Ω_1_*, *Ω_2_*, the phases of the rotor imbalances, respectively.

A rigid rotor can be balanced in two different planes [8]. The schematic diagram of the two-plane balancing method is shown in Figure 9, where *r_b_* is the radius of the balancing plane, *l_b_* is the distance between the balancing planes, *m_ε_* and *m_σ_* are the correction masses to compensate the static and dynamic imbalances, respectively.

According to the equivalent formula, the correction masses are given by:

(18) $$\left\{ \begin{array}{l} m_{\varepsilon }=\frac{m\varepsilon }{2r_{b}}\\ m_{\sigma }=\frac{\left(J_{z}-J_{r}\right)\sigma }{r_{b}l_{b}} \end{array}\right.$$

Two or four screws with values, which match *m_ε_* and *m_σ_* and can be added or resolved in the vector form are fixed in the balancing holes to balance the rotor [28].

## 5. Suppression of the Harmonic Vibrations Induce by the Sensor Runout

**Figure 10:**
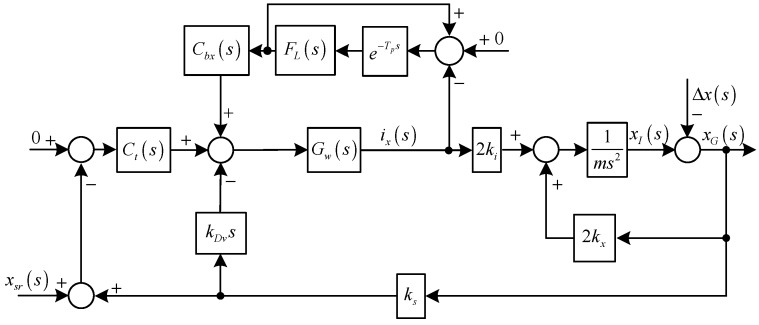
Diagram of the translational subsystem in the *X* direction with the repetitive controller.

Theoretically, only the sensor runout remains if the field balancing is well achieved. Then the harmonic vibrations (torque and force) can be suppressed by reducing harmonic currents, which are induced by the sensor runout only related to *k_i_*. A plug-in repetitive control method is employed owing to its ability to reduce all the harmonic currents simultaneously with a light computational load. The repetitive controller is utilized in a feedback form, because *G_w_*(*s*) is voltage-sourced. The diagram of the translational subsystem in the *X* direction with the repetitive controller is shown in Figure 10, where *T_p_* is the dead time, *F_L_*(*s*) is a fist-order low-pass filter, *C_bx_*(*s*) is a lead element to compensate the phase lad due to the power amplifiers and to improve the system bandwidth, and:

(19) $$\left\{ \begin{array}{l} F_{L}\left(s\right)=\frac{\omega_{L}}{s+\omega_{L}}\\ C_{bx}\left(s\right)=k_{cx}\frac{s+\omega_{w}}{k_{\omega x}s+\omega_{w}} \end{array}\right.$$

where *ω_L_* is the cut-off frequency, *k_cx_* and *k_wx_* are positive parameters to be chosen. 

The suppression factor of the sensitivity function due to the repetitive controller is given by [33]:

(20) $$M_{x}\left(s\right)=\frac{1-F_{L}\left(s\right)e^{-T_{p}s}}{1-\left(1-\frac{\left(ms^{2}-2k_{x}\right)C_{bx}\left(s\right)G_{w}\left(s\right)}{ms^{2}-2k_{x}+2k_{i}k_{s}G_{w}\left(s\right)\left(C_{t}\left(s\right)+k_{Dv}s\right)}\right)F_{L}\left(s\right)e^{-T_{p}s}}$$

To suppress the harmonic currents, let:

(21) $$\underset{s=j2n\pi /T_{p}}{\lim}\left|M_{x}\left(s\right)\right|=0$$

We have:

(22) $$\left\{ \begin{array}{l} \underset{s=j2n\pi /T_{p}}{\lim}\left|F_{L}\left(s\right)\right|=1\\ \underset{s=j2n\pi /T_{p}}{\lim}∠F_{L}\left(s\right)e^{-T_{p}s}=0 \end{array}\right.$$

To satisfy the gain and phase requirements, $\omega_{L}>2n_{m}\pi /T_{p}$, *n_m_* is the largest number of the harmonic to be suppressed. *T_p_* is set with:

(23) $$T_{p}=\frac{2\pi }{\Omega }\left[1-\frac{1}{2\pi }\tan^{-1}\left(\frac{\Omega }{\omega_{L}}\right)\right]$$

To compensate the phase lag of all the suppressed harmonics due to the power amplifiers, $k_{\omega p}<\omega_{w}T_{p}/2n_{m}\pi$. If *k_cx_* = 0, the repetitive controllers will be closed. *k_cx_* is closely related with the system characteristics. It can be chosen according to the stability analysis and the performances of the repetitive controllers in simulations and experiments with the increase of the value from 0.

The characteristic equation of the translational subsystem with a single time delay *T_p_* can be expressed by:

(24) $$P_{x}\left(s\right)+Q_{x}\left(s\right)e^{-T_{p}s}=0$$

where:

$$\left\{ \begin{array}{l} P_{x}\left(s\right)=\left\{2k_{i}k_{s}k_{w}\omega_{w}s\left[C_{t}\left(s\right)+k_{Dv}s\right]+\left(ms^{3}-2k_{x}s\right)\left(s+\omega_{w}\right)\right\}\left(k_{\omega x}s+\omega_{w}\right)\left(s+\omega_{L}\right)\\ Q_{x}\left(s\right)=\omega_{L}\left(k_{cx}k_{w}\omega_{w}-k_{\omega x}s-\omega_{w}\right)\left(ms^{3}-2k_{x}s\right)\left(s+\omega_{w}\right)-2k_{i}k_{s}k_{w}\omega_{w}\omega_{L}s\left(k_{\omega x}s+\omega_{w}\right)\left[C_{t}\left(s\right)+k_{Dv}s\right] \end{array}\right.$$

The regeneration spectrum for this system is defined as:

(25) $$R_{x}\left(\omega \right)=\left|\frac{Q_{x}\left(\text{j}\omega \right)}{P_{x}\left(\text{j}\omega \right)}\right|$$

The absolute stability can be guaranteed if:

1. The polynomial has no zeros in the right half of the *s*-plane.
2. $R_{x}\left(\omega \right)<1,\;\forall \omega \in R_{+}$.

**Figure 11:**
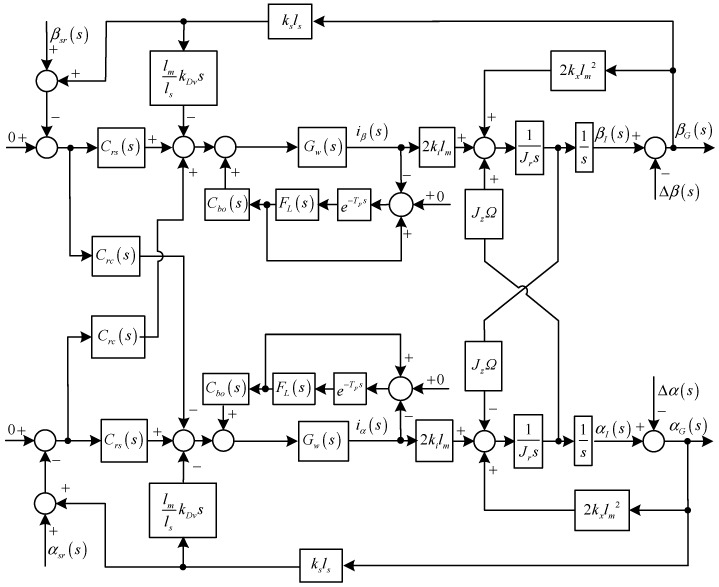
Diagram of the rotational system with the repetitive controllers.

Similarly, repetitive controllers are designed in the rotational subsystem, as shown in Figure 11, where:

(26) $$C_{bo}\left(s\right)=k_{co}\frac{s+\omega_{w}}{k_{\omega x}s+\omega_{w}}$$

where *k_co_* is the gain, and *k_co_* ≥ 0.

## 6. Simulations and Experiments

**Figure 12:**
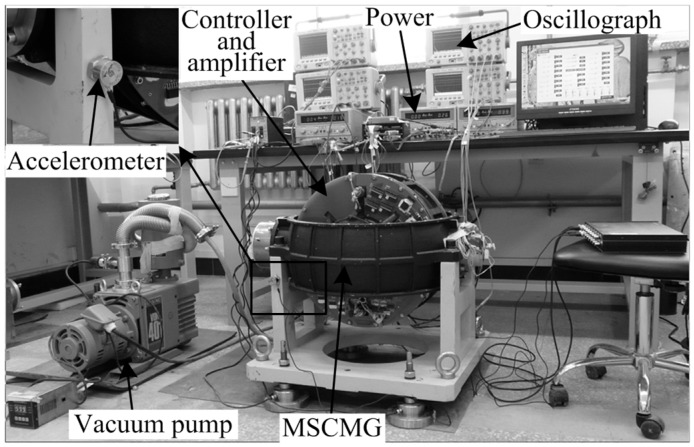
The photograph of the magnetically-suspended control moment gyro experimental rig.

To demonstrate the effectiveness of the proposed control methods, comparative simulations and experiments have been developed. Figure 12 shows the experimental rig of the magnetically-suspended control moment gyro (MSCMG), which consists of a gimbal system and a high-speed rotor system with AMBs. The high-speed rotor system with AMBs is very suitable to verify the proposed field balancing and harmonic vibration suppression methods. The experiment setup is composed of vacuum pump, power, controller and amplifier, accelerometer, oscillographs, and MSCMG. A high-speed rigid and flat AMB-rotor is inside the MSCMG room. The vacuum pump is employed to create a nearly vacuous environment (the air pressure is about 2 Pa) in the MSCMG room, in which the magnetically-suspended rotor is driven by the controller and amplifier. The proposed control algorithm is implemented in a digital signal processor-based controller with a sampling and control period of 150 μs. Eight eddy-current sensors and one Hall sensor are employed to measure the displacements of the geometric axis and the relative angular positions to the reference point, respectively. An accelerometer is employed to measure the harmonic vibration acceleration transmitted to the support bracket, which is fixed on the spacecraft in space. The measured signals of the harmonic displacements, currents, and vibrations are shown on the oscillographs. The nominal speed of the AMB-rotor system is 200 Hz. It is noted that the first, third, and fifth harmonics are dominant components in actual experiments; therefore, the harmonic frequencies are 200, 600, and 1000 Hz.

The parameters of the AMB system are chosen to match those in the experimental rig, presented in Table 1. The rotor is designed to be flat (As shown in Table 1, $J_{z}/J_{r}\approx 1.32$) for a large moment of inertia. To simulate the actual current noises and to test the performance of the proposed control methods at all the frequencies, a random noise with the mean and variance values of 0 and 3 × 10^−4^, respectively, is injected into the currents in simulations.

**Figure 13:**
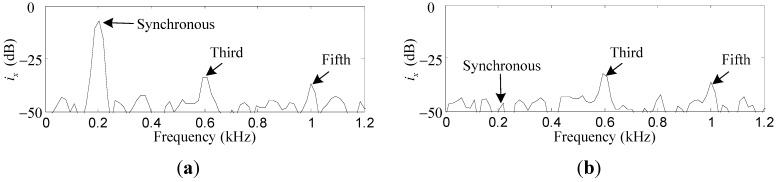
Simulation results of *i_x_*: (**a**) before synchronous current reduction; (**b**) after synchronous current reduction.

**Figure 14:**
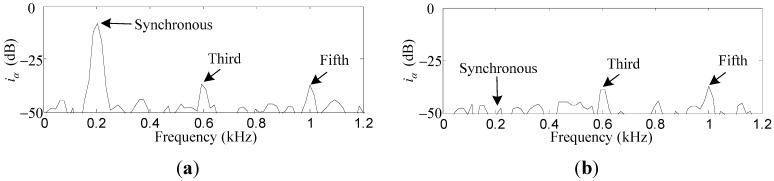
Simulation results of *i_α_*: (**a**) before synchronous current reduction; (**b**) after synchronous current reduction.

Comparative simulation results of *i_x_* and *i_α_* before and after the synchronous current reduction are presented. As shown in Figure 13 and Figure 14, the synchronous *i_x_* and *i_α_* are reduced by 41.2 dB and 40.7 dB, respectively, if the variable-phase notch feedback block is activated. 

**Table 1:** Parameters of the AMB system with the proposed control approach.

Parameters	Values	Parameters	Values
*m*	57 kg	*θ_n_*	0.94 rad
*J_r_*	0.62 kg∙m^2^	*ω_L_*	10^4^ rad/s
*J_z_*	0.82 kg∙m^2^	*T_p_*	0.0049 s
*l_m_*	0.113 m	*k_cx_*	960
*l_s_*	0.178 m	*k_ωx_*	0.1
*k_i_*	450 N/A	*k_co_*	1100
*k_x_*	2.5 × 10^6^ N/m	*ε*	5 × 10^−6^ m
*k_s_*	1.5 × 10^7^ V/m	*χ*	π/3 rad
*ω_w_*	1683 rad/s	*σ*	2.8 × 10^−5^ rad
*k_w_*	1.23 × 10^−4^ A/V	*δ*	−π/3 rad
*r_b_*	0.21 m	*s_as1_*	10
*l_b_*	0.07 m	*α_s1_*	π/4 rad
*k_P_*	5	*s_as3_*	4
*k_I_*	40	*α_s3_*	4π/3 rad
*k_IM_*	0.05	*s_as5_*	1
*k_D_*	0.01	*α_s5_*	9π/5 rad
*k_rh_*	0.01	*s_bs1_*	12
*k_rl_*	0.001	*β_s1_*	5π/3 rad
*ω_rh_*	1256.6 rad/s	*s_bs3_*	5
*ω_rl_*	314.2 rad/s	*β_s3_*	11π/6 rad
*ϕ*	2.5 rad	*s_bs5_*	2
*φ*	0.9 rad	*β_s5_*	π/5 rad
*ξ_n_*	2 × 10^6^		

**Figure 15:**
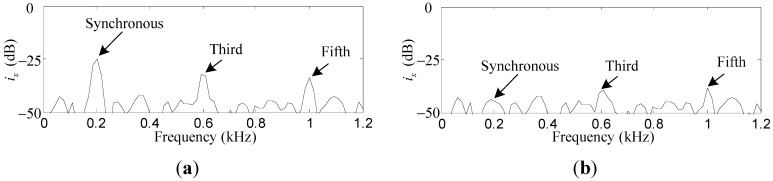
Simulation results of *i_x_*: (**a**) before harmonic current suppression; (**b**) after harmonic current suppression.

**Figure 16:**
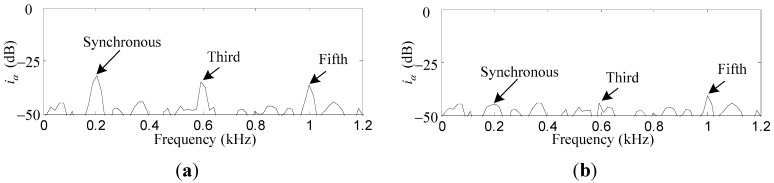
Simulation results of *i_α_*: (**a**) before harmonic current suppression; (**b**) after harmonic current suppression.

**Figure 17:**
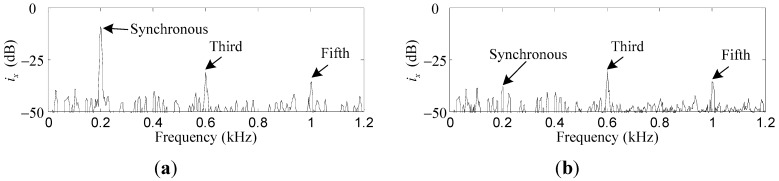
Experiment results of *i_x_*: (**a**) before synchronous current reduction; (**b**) after synchronous current reduction.

To verify the suppression effect of the harmonic *i_x_* and *i_α_* induced by the sensor runout, the repetitive controller is enabled. As can be seen in Figures 15a,b, the first, third, and fifth harmonics of *i_x_* are suppressed by 18.8 dB, 7.3 dB and 4.7 dB, respectively. Similar results can be found in Figures 16a,b, where the first, third, and fifth harmonics of *i_α_* are suppressed by 12.7 dB, 8.9 dB, and 4.5 dB, respectively. The harmonics of *i_x_* and *i_α_* are suppressed by a considerable amount, although the attenuation degree decreases with the increase of the harmonic number due to the low-pass *F_L_*(*s*).

**Figure 18:**
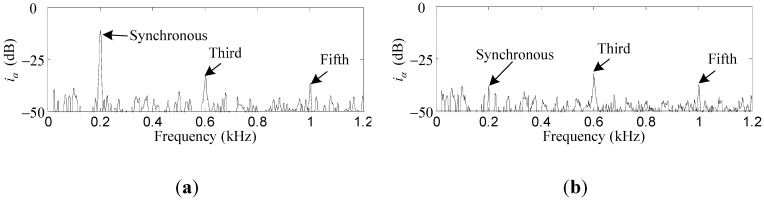
Experiment results of *i_α_*: (**a**) before synchronous current reduction; (**b**) after synchronous current reduction.

Experiments of the synchronous currents reduction at varied speeds of 180 and 200 Hz were carried out for field balancing. Only the experimental results of 200 Hz are shown in accord with those of the simulations. As shown in Figure 17 and Figure 18, the synchronous *i_x_* and *i_α_* were reduced by 26.2 dB and 27.1 dB, respectively. The correction masses were calculated as $m_{\varepsilon }\text{ = }0.681$ g and $m_{\sigma }\text{ = }0.383$ g. 

**Figure 19:**
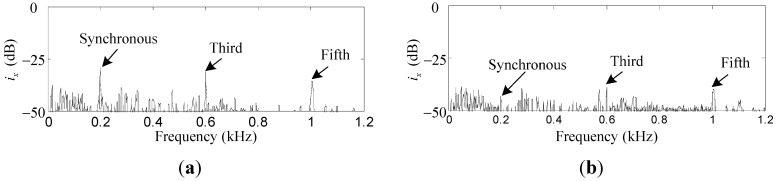
Experiment results of *i_x_*: (**a**) before harmonic current suppression; (**b**) after harmonic current suppression.

**Figure 20:**
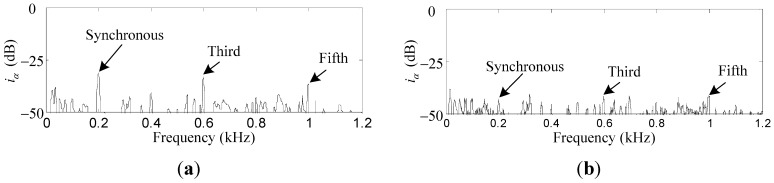
Experiment results of *i_α_*: (**a**) before harmonic current suppression. (**b**) after harmonic current suppression.

Compared Figure 19a and Figure 20a with Figure 17a and Figure 18a, the synchronous currents are much smaller after the field balancing. Hence, the rotor imbalances are well compensated. To further suppress the harmonic vibrations, the harmonic currents induced by the sensor runout were attenuated by the repetitive controllers. As shown in Figure 19 and Figure 20, the first, third, and fifth harmonic currents of *i_x_* (*i_α_*) were suppressed by 12.0 dB, 6.9 dB, and 3.6 dB (11.1 dB, 8.5 dB, and 4.3 dB), respectively. Good matching between the experiment results and the simulation results was achieved.

**Figure 21:**
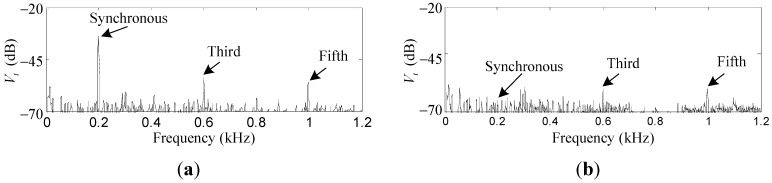
Experiment results of *V_t_*: (**a**) before field balancing and harmonic vibration suppression; (**b**) after field balancing and harmonic vibration suppression.

The measured acceleration *V_t_* by the accelerometer is used to demonstrate the overall effects of the proposed field balancing and harmonic vibration suppression methods. Figure 21 shows the values of first, third, and fifth harmonic vibrations are reduced by 31.1 dB, 6.8 dB, and 3.9 dB, to similar sizes to those of noises (mainly caused by the gyroscopic effects and structural resonance), which means the rotor imbalance is well compensated and the harmonic vibrations are significantly suppressed.

## 7. Conclusion

In this work, field balancing and harmonic suppression in AMB with both rotor imbalances and sensor runout are studied. The variable-phase notch feedback is used to reduce the synchronous currents, and then rotor imbalances are identified and compensated according to the synchronous displacement relationships of the geometric, inertial, and rotational axes of the rotor. To further reduce the harmonic currents induced by the sensor runout, a repetitive control method is proposed. Both simulations and experiments are performed to verify the proposed methods. The measured acceleration is employed to demonstrate the overall effect of the field balancing and vibration suppression. The experiment results show the first, third, and fifth harmonics are reduced by 31.1 dB, 6.8 dB, and 3.9 dB, respectively, and no visible mutual couplings between the harmonics and the other frequencies exist. This means the field balancing and harmonic vibration suppression are well achieved, and the frequencies other than the harmonics of the vibrations are not affected by the proposed methods. The field balancing is performed during a speed up from 180 Hz to 200 Hz. It is suitable for rigid rotors, since the rotor imbalances change little over the full range of the rotor speed, once the rotor imbalance identification is completed. The proposed strategies are suitable for high-precision and micro-vibration applications with AMB devices, such as space actuators (e.g. control moment gyros and flywheels). Further research work will focus on the robustness of the field balancing and harmonic vibration suppression methods.
